# CaSilico: A versatile CRISPR package for *in silico* CRISPR RNA designing for Cas12, Cas13, and Cas14

**DOI:** 10.3389/fbioe.2022.957131

**Published:** 2022-08-09

**Authors:** Adnan Asadbeigi, Milad Norouzi, Mohammad Sadegh Vafaei Sadi, Mojtaba Saffari, Mohammad Reza Bakhtiarizadeh

**Affiliations:** ^1^ Department of Medical Genetics, Cancer Institute, Faculty of Medicine, Tehran University of Medical Sciences (TUMS), Tehran, Iran; ^2^ Department of Bioinformatics, Institute of Biochemistry and Biophysics, University of Tehran, Tehran, Iran; ^3^ Department of Medical Genetics, Faculty of Medicine, Tehran University of Medical Sciences (TUMS), Tehran, Iran; ^4^ Department of Animal and Poultry Science, College of Aburaihan, University of Tehran, Tehran, Iran

**Keywords:** gene editing, genome engineering, Cas13, Cas12, guide RNA

## Abstract

The efficiency of the CRISPR-Cas system is highly dependent on well-designed CRISPR RNA (crRNA). To facilitate the use of various types of CRISPR-Cas systems, there is a need for the development of computational tools to design crRNAs which cover different CRISPR-Cas systems with off-target analysis capability. Numerous crRNA design tools have been developed, but nearly all of them are dedicated to design crRNA for genome editing. Hence, we developed a tool matching the needs of both beginners and experts, named CaSilico, which was inspired by the limitations of the current crRNA design tools for designing crRNAs for Cas12, Cas13, and Cas14 CRISPR-Cas systems. This tool considers a comprehensive list of the principal rules that are not yet well described to design crRNA for these types. Using a list of important features such as mismatch tolerance rules, self-complementarity, GC content, frequency of cleaving base around the target site, target accessibility, and PFS (protospacer flanking site) or PAM (protospacer adjacent motif) requirement, CaSilico searches all potential crRNAs in a user-input sequence. Considering these features help users to rank all crRNAs for a sequence and make an informed decision about whether a crRNA is suited for an experiment or not. Our tool is sufficiently flexible to tune some key parameters governing the design of crRNA and identification of off-targets, which can lead to an increase in the chances of successful CRISPR-Cas experiments. CaSilico outperforms previous crRNA design tools in the following aspects: 1) supporting any reference genome/gene/transcriptome for which an FASTA file is available; 2) designing crRNAs that simultaneously target multiple sequences through conserved region detection among a set of sequences; 3) considering new CRISPR-Cas subtypes; and 4) reporting a list of different features for each candidate crRNA, which can help the user to select the best one. Given these capabilities, CaSilico addresses end-user concerns arising from the use of sophisticated bioinformatics algorithms and has a wide range of potential research applications in different areas, especially in the design of crRNA for pathogen diagnosis. CaSilico was successfully applied to design crRNAs for different genes in the SARS-CoV-2 genome, as some of the crRNAs have been experimentally tested in the previous studies.

## 1 Introduction

Coevolution between prokaryotes and predators (such as viruses) has resulted in a large number of natural defense mechanisms to provide immunity against their enemies. In this context, some of the intracellular regulatory and defense mechanisms are based on complementary nucleic acid base-pairing to regulate or destroy the genetic targets. One of these mechanisms is the CRISPR-Cas (clustered regularly interspaced short palindromic repeats-CRISPR-associated protein) system, which is an adaptive and heritable prokaryotic immune system that provides acquired immunity against the invasive phage infection. After the first adaption of the CRISPR-Cas system for genome editing in 2012, this technology has revolutionized genetic engineering technology and has been considered a pioneer in effective genome and transcriptome editing (Ishino et al., 2018). Based on the most recent classification of CRISPR-Cas systems, there are two classes subdivided into six types and 33 subtypes, which differ in effector proteins and composition of the Cas genes (Makarova et al., 2020). Class I contains types I, III, and IV uses multiple proteins as an effector to mediate interference, while class II containing types II, V, and VI has a simple architecture composed of only one Cas multi-domain effector protein (Pickar-Oliver and Gersbach, 2019). Of these variants, only a few Cas proteins (from class II) have the potential to edit genome/gene/transcriptome such as Cas9, Cas12, Cas13, and Cas14.

The success of a CRISPR experiment, targeting specificity and cleavage efficiency, mainly depends on the choice of the best suited CRISPR RNA (crRNA) for an appropriate target site and with minimal off-target effects (Nidhi et al., 2021). Over the past years, numerous computational programs have been developed to design optimal crRNAs required for Cas protein targeting a specific region in various species (Li et al., 2022). A list of widely used crRNA design tools is presented in Table 1. Also, a detailed list of the developed crRNA design tools is provided in Supplementary Table S1. Despite this rapidly growing list of tools, most of the current crRNA tools only support Cas9 type, and a few number of tools are developed to design crRNAs for other types of CRISPR-Cas systems (like Cas12 or Cas13) such as CHOPCHOPv3, CRISPOR, and Cas13design (Concordet and Haeussler, 2018; Labun et al., 2019; Wessels et al., 2020). Moreover, these tools have some disadvantages such as limitation to a pre-defined and restricted list of genomes. Some tools present more flexibility incorporating any genome provided by users, but they lack the flexibility to cover most of the Cas protein subtypes, like Cas13design that only supports subtype D of type VI CRISPR-Cas system (Wessels et al., 2020). It is worth to note that none of the current tools consider a comprehensive list of the principal rules to design crRNA for types VI and V-F1. On the other hand, the available tools have to be updated to include the continuous development of new types or subtypes of CRISPR-Cas systems.

**TABLE 1:** Summary of commonly used crRNA design tools.

Tool	Cas enzyme	Species support	Sequence ID* search	Off-target search	Result visualization	Reference
CRISPOR	Cas9 and Cas12 variants	Many	No	Yes	Yes	Concordet and Haeussler, (2018)
CHOPCHOP	Cas9, Cas12, and Cas13 variants	Many	Yes	Yes	Yes	Labun et al. (2019)
Cas13design	Cas13d variant	Many	Yes	Yes	No	Wessels et al. (2020)
E-CRISP	SpCas9 variant	Many	Yes	Yes	No	Heigwer et al. (2014)
CRISPick	SpCas9, SaCas9, AsCas12a, and enAsCas12a variants	Human, mouse, and rat	Yes	Yes	No	Doench et al. (2014)
GUIDES	SpCas9 variant	Human and mouse	Yes	Yes	Yes	Meier et al. (2017)
Microsoft Research CRISPR	SpCas9 variant	Human	Yes	Yes	No	Listgarten et al. (2018)
Cas-OFFinder	Cas9 and Cas12 variants	Many	No	Yes	No	Bae et al. (2014)
Off-Spotter	SpCas9, CjCas9, and SaCas9 variants	Human, mouse, and yeast	No	Yes	Yes	Pliatsika and Rigoutsos, (2015)

Nowadays, there is a huge interest in transcript targeting, nucleic acid detection, and applying new approaches for vaccination by Cas13. Also, the miniature nature of Cas14 enzymes makes them a good tool for SNP detection. Integrating all rules of designing crRNA into an automated highly versatile tool enables researchers to easily and efficiently design effective crRNAs for particular experiments. CaSilico considers multiple criteria for crRNA designing including mismatch tolerance rules, self-complementarity, GC content, frequency of cleaving base around the target site, target accessibility, and PFS (protospacer flanking site) or PAM (protospacer adjacent motif) requirement. It also predicts specificity of each candidate by employing restricted off-target search algorithms and represents all results in an interactive graphical interface. To increase the flexibility, our software allows users to modify several parameters such as conservation threshold and the method of identifying conserved windows. Sequence variations in transcripts of a gene or different strains of a virus make it difficult to design efficient crRNAs, which match all the related sequences. CaSilico can automatically identify conserved regions in these sequences and use them as target for crRNA designing. In this study, we developed a powerful and user-friendly R package, named CaSilico, to facilitate in silico crRNA designing for different subtypes of Cas13 and Cas12 systems. To showcase CaSilico functionalities, it was applied to design specific crRNAs for detecting different strains of severe acute respiratory syndrome coronavirus 2 (SARS-CoV-2), as a case study. The suggested crRNAs can be considered as potential candidates for the detection of the relevant viruses using CRISPR-Cas-based diagnostics.

## 2 Materials and methods

### 2.1 The CaSilico input

CaSilico accepts three kinds of input formats as target sequences to be scanned for all potential target sites: 1) direct sequence(s) (genome, gene, or transcript), 2) genome/gene/transcript identifier(s) (RefSeq or Ensembl ID), and 3) genomic coordinate. There is no limitation on size of the input sequence for analysis as observed in some of the current tools such as Cas13design. When a user enters sequence ID, the tool connects to the GenBank database and retrieves the sequence corresponding to that ID via “read.GenBank” function from ape package (Paradis and Schliep, 2019).

### 2.2 Supported CRISPR-Cas types/subtypes

**FIGURE 1:**
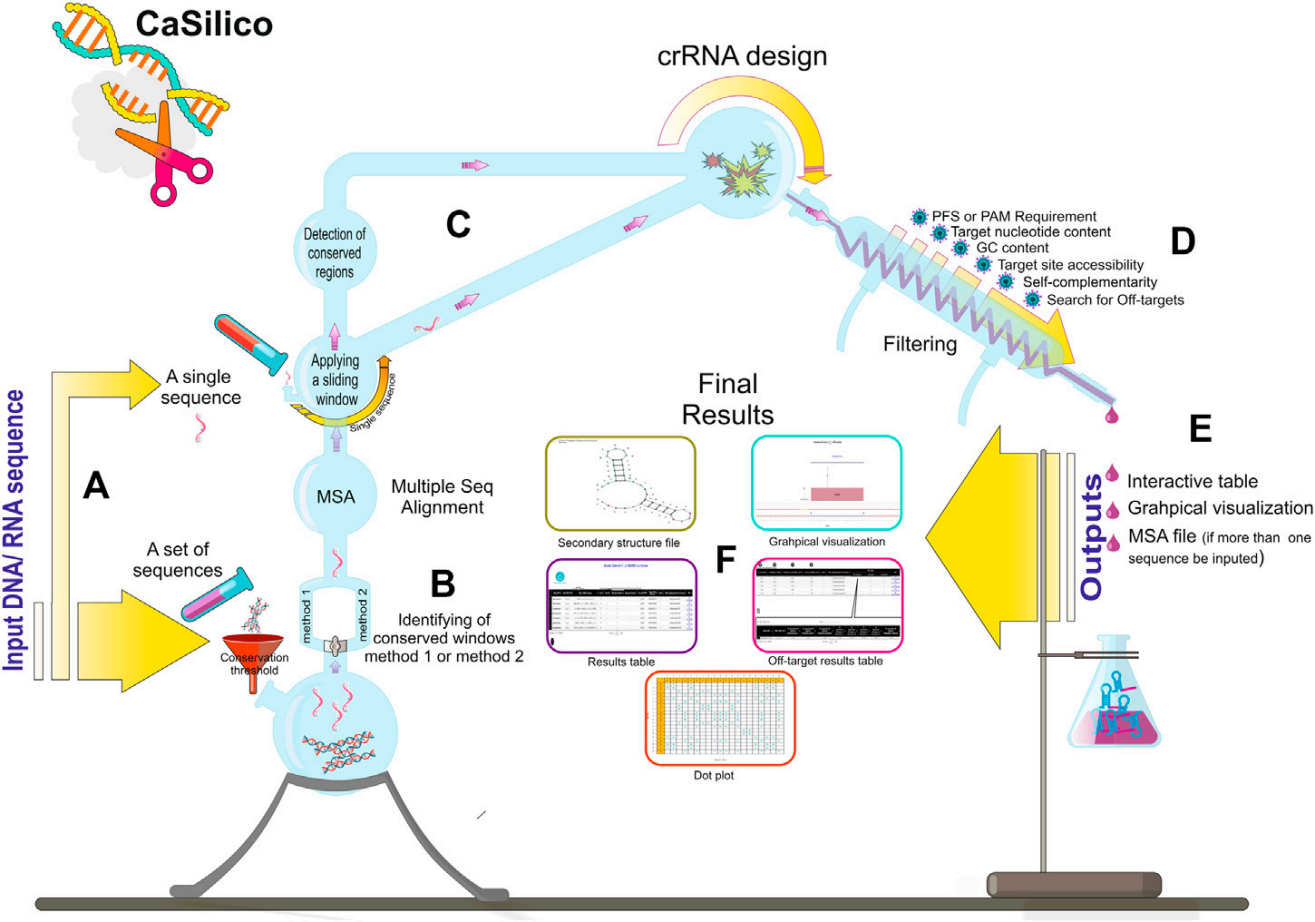
CaSilico workflow. **(A)** CaSilico accepts a single or a set of DNA or RNA sequences to be scanned for crRNA designing. **(B)** When more than one sequence is given as input, the conserved regions among them are automatically detected considering conservation threshold and one of the two different approaches for identifying conserved regions. **(C)** A sliding window (stride of 1 nt) is employed across the single sequences or conserved region of multiple sequences to specify potential target sites. **(D)** CaSilico applies multiple criteria for crRNA designing, performs off-target analysis, and returns outputs in an interactive graphical interface and some files such as MSA and secondary structure **(E,F)**.

Class 2 types VI and V CRISPR-Cas systems include different subtypes with various Cas proteins; however, LwaCas13a, PspCas13b, and RfxCas13d proteins from type VI-A/B/D and LbCas12a, AapCas12b, and Cas14a proteins from type V-A/B/F1 are of particular interest to researchers since they have highest efficiency as was found in previous studies (Table 2) (Shmakov et al., 2015; Abudayyeh et al., 2017; Cox et al., 2017; Gootenberg et al., 2017; Smargon et al., 2017; Chen et al., 2018; Harrington et al., 2018; Konermann et al., 2018; Teng et al., 2018; Wessels et al., 2020). Hence, CaSilico, a comprehensive and novel computational pipeline, was developed to design crRNAs for type VI-A/B/D and type V-A/B/F1 CRISPR-Cas systems, as outlined in Figure 1. The user only needs to select the CRISPR type of interest using CRISPRTypes argument.

**TABLE 2:** Table shows class 2 CRISPR-Cas system variants for which CaSilico designs crRNA.

CRISPR-cas system	Cas protein type	Corresponding organism	Application
VI-A	Cas13a/C2c2	*Leptotrichia wadei*	Nucleic acid detection, vaccination, transcript targeting, and SNP detection
VI-B	Cas13b/C2c6	Prevotella sp. P5-125	Nucleic acid detection, vaccination, transcript targeting, and SNP detection
VI-D	Cas13d	*Ruminococcus flavefaciens* XPD3002	Nucleic acid detection, vaccination, transcript targeting, and SNP detection
V-A	Cas12a/Cpf1	Lachnospiraceae bacterium ND2006	Nucleic acid detection and genome editing
V-B	Cas12b/C2c1	*Alicyclobacillus acidiphilus*	Nucleic acid detection and genome editing
V-F1	Cas14a/Cas12f1	Uncultured archaea	SNP detection

**FIGURE 2:**
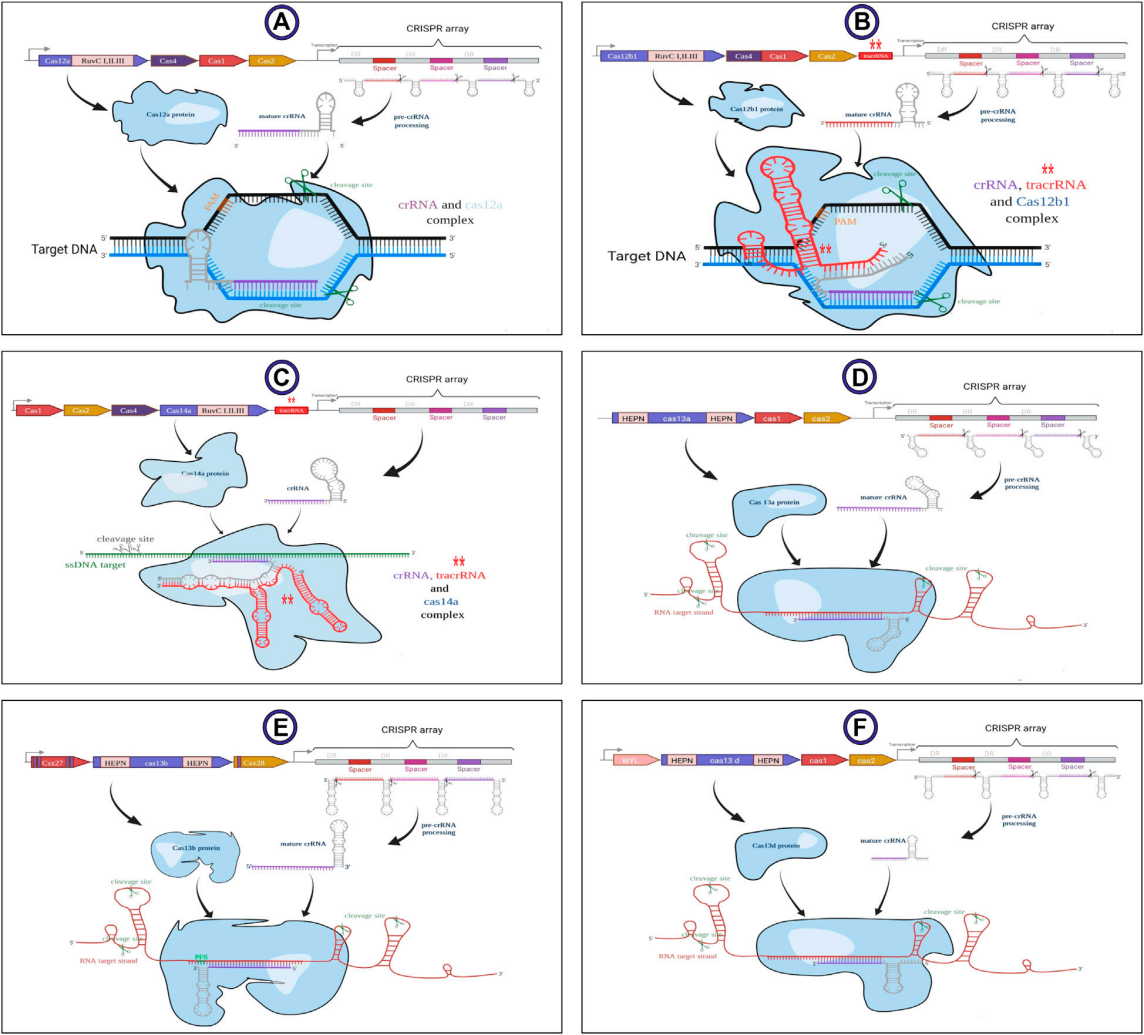
Schematic structure of the subtypes of V and VI types related to class II of CRISPR-Cas system. Generalized locus along with their main details illustrated in sections **(A)** V-A, **(B)** V-B1, **(C)** V-F1, **(D)** VI-A, **(E)** VI-B, and **(F)** VI-D. LwaCas13a requires spacers of at least 20 nt length but shorter spacers have lower efficiency. AapCas12b and Cas14a are dual-RNA-guided endonucleases that require association with a structural accessory RNA named transactivating CRISPR RNA (tracrRNA) for their activity. This image was created using www.biorender.com.

The structure of crRNA differs in several properties in various CRISPR-Cas systems. The schematic structure of all covered subtypes by CaSilico is displayed in Figure 2. Taking these differences into account, the crRNAs were designed as 36 nt unprocessed DR in LwaCas13a and PspCas13b, while a 30 nt processed DR was considered in RfxCas13d (changing the first base pair of DR (A to T) increases Cas13d targeting efficacy). On the other hand, the size of mature spacers for LwaCas13a, PspCas13b, and RfxCas13d are considered as 28, 30, and 22 nt, respectively. The size of DR in type V-A/B/F1 CRISPR locus was defined as 21, 22, and 35 nt, respectively, while guide possesses a size of 20 nt for all these subtypes. Since AapCas12b and Cas14a are dual-RNA-guided endonucleases, sgRNA for Cas14a is considered as the crRNA/tracrRNA (transactivating CRISPR RNA) duplex and a chimeric sgRNA was designed by the fusion of crRNA/tracrRNA duplex for AapCas12b.

### 2.3 Principles of crRNA designing

Since crRNA structures of different subtypes of CRISPR-Cas system have their specific features, different rules and parameters have to be taken into account to design crRNA for each subtype. For example, PAM/PFS sequence, PAM/PFS orientation, and protospacer length vary across different types and subtypes of CRISPR-Cas systems. Hence, based on previous experiments and data generated by different researchers, various crRNA design rules have been proposed. A deep literature mining enabled us to collect these features. These key features for Cas12 and Cas13 are summarized in Tables 3 and 4, respectively. To accommodate different Cas proteins with various specificity requirements, these features were considered and integrated by CaSilico to design crRNA for each subtype. In order to achieve this, in consideration of the fact that the first step in designing a crRNA is identifying a targetable sequence space, CaSilico lists all the potential target sites within the input sequence by considering PAM/PFS sequence requirement and crRNA length. If more than one sequence is input, pipeline begins with detecting conserved regions in the sequences according to the user-defined conservation thresholds. Each identified candidate crRNA is then evaluated according to the rules in Tables 3 and 4. In the case of type V-A/B CRISPR-Cas systems, both forward and reverse strands of the target sequence are searched for potential crRNAs.

**TABLE 3:** Principles of crRNA designing for type VI-A/B/D CRISPR-Cas system.

Rule No	Parameter	
	Positional rule	
**1**	Perfect base pairing with target region^a^	
1–1	Allowing single mismatches into the spacer	
	Type VI-A	Central (seed) region or base pairs 13 to 24 LwaCas13a target are intolerant to single mismatches
	Type VI-B	Central (seed) region or base pairs 12 to 26 PspCas13b target are intolerant to single mismatches
	Type VI-D	Central (seed) region or base pairs 2 to 8 RfxCas13d target are intolerant to single mismatches
1–2	Allowing consecutive or nonconsecutive double mismatches into the spacer	
	Type VI-A	Central (seed) region or base pairs 8 to 27 LwaCas13a target are intolerant to double mismatches (mismatch occurrence exactly at 5′ or 3′ end is preferred)
	Type VI-B	Central (seed) region or base pairs 12 to 29 PspCas13b target are intolerant to double mismatches (mismatch occurrence exactly at 5′ or 3′ end is preferred)
	Type VI-D	Double mismatches exactly at 5′ or 3′ end are acceptable
**2**	Protospacer flanking site or sequence (PFS) requirement	
	LwaCas13a lacks PFS.	
	In PspCas13b, not being D (G, A, or U) at first and second sites of 5′ PFS inhibits single-stranded RNA cleavage and being NAN or NNA at 3′ PFS enhances this activity	
	The PFS may not be necessary for RfxCas13d	
**3**	Target nucleotide content	
	Cleavage preferentially occurs in uracil-rich regions (poly UU/AU; at uracil bases) for LwaCas13a and RfxCas13d and in adenine-rich regions (at adenine bases) for PspCas13b	
**4**	GC content	
Thermodynamics rules		
**5**	Target site accessibility	
	Not having any stable secondary structures in target region. This feature is important in type VI-A/B/D that targets RNA	
**6**	Self-complementarity	
BLAST rules		
**7**	Searching off-target effects in target organism	
**8**	Searching off-target effects in other organisms	

a G-U wobble base pairing is allowable.

**TABLE 4:** Principles of crRNA designing for type V-A/B/F1 CRISPR-Cas system.

Rule no	Parameters	
	Positional rules	
**1**	Perfect base pairing with target region^a^	
1–1	Allowing single and consecutive or nonconsecutive double mismatches into the spacer	
	Type V-A	Central (seed) region or base pairs 1 to 6 LbCas12a target are intolerant to single and consecutive or nonconsecutive double mismatches
	Type V-B	Single and consecutive or nonconsecutive double mismatches at any positions of AapCas12b target are acceptable
	Type V-F1	Central (seed) region or base pairs 9 to 16 Cas14a target are intolerant to single and consecutive or nonconsecutive double mismatches
**2**	Protospacer adjacent motif (PAM; CRISPR motif) requirement	
	PAM sequence of LbCas12a and AapCas12b for target DNA recognition is 5′-TTTV-3′ and 5′-TTN-3′, respectively, located upstream of the target sequence. PAM is not necessary for Cas14a	
**3**	GC content	
Thermodynamics rules		
**4**	Target site accessibility	
	Not having any stable secondary structures in target region. This feature is important in type V-F1 that targets ssDNA.	
**5**	Self-complementarity	
BLAST rules		
**6**	Searching off-target effects in target organism	
**7**	Searching off-target effects in other organisms	

a G-U wobble base pairing is allowable.

### 2.4 PAM/PFS requirement

Protospacer adjacent motif (PAM) or protospacer flanking sequence (PFS, in type VI) is a short motif directly adjacent to the target sequence. Cas protein can be activated to cleave the target nucleic acid if both protospacer and PAM/PFS sites are present in the target (Leenay and Beisel, 2017). Some Cas proteins are PFS- or PAM-independent, and this independency increases the availability of target sites, while the others recognize a unique PFS or PAM motif that its recognition limits eligible regions of a target sequence (Shmakov et al., 2015; Abudayyeh et al., 2016, 2017; Cox et al., 2017; Chen et al., 2018; Konermann et al., 2018; Teng et al., 2018; Pickar-Oliver and Gersbach, 2019). CaSilico filters the potential target sites for each Cas protein based on PAM or PFS requirements according to Tables 3 and 4.

### 2.5 Target nucleotide content

In type VI CRISPR-Cas system, Cas13–crRNA complex recognizes and binds to complementary ssRNA targets and then cleaves ssRNA with a potential preference for certain nucleotides. Hence, the presence of specific nucleotides at certain locations of the target RNA may influence Cas13 nuclease activity. It is well known that LwaCas13a and RfxCas13d in complex with their crRNAs bind to complementary target sequence and preferentially cleave it at uracil-rich regions; however, PspCas13b exhibits a significant preference for adenine-rich regions (Table 3) (Shmakov et al., 2015; Abudayyeh et al., 2016; East-Seletsky et al., 2017; Gootenberg et al., 2018; Freije et al., 2019; Wessels et al., 2020). With this in mind, nucleotide content of the potential target sites was calculated as the proportion of uracils or adenines (depending on chosen subtype) in 100 nt upstream and downstream of the target site, called local U/A-rich. The local U/A-rich was normalized by the proportion of uracils or adenines throughout the gene to create normalized U/A-rich. When more than one sequence is given as input, this parameter can be estimated based on the consensus sequence, which is obtained based on the MSA results, so that the allele frequencies of uracils or adenines are divided by allele frequencies of all bases in 100 nt windows upstream and downstream of spacer site. The local U/A-rich parameter enables us to identify more efficient crRNAs since a higher value of this parameter is preferred.

### 2.6 GC content

The binding efficiency of the crRNA may be influenced by certain criteria such as guanine–cytosine content (GC-content). Previous studies suggested that crRNAs are most effective with a GC-content in the range of 40–70% (Wang et al., 2014; Tsai et al., 2015). CaSilico provides the information of GC content for each crRNA to be considered.

### 2.7 Target site accessibility

It is well known that target site accessibility influences crRNA functionality for RNA targeting since accessible sites can better interact with the cognate crRNA (Bandaru et al., 2020). Therefore, target site accessibility has to be considered while designing crRNA in types VI-A/B/D and V-F1 to develop an accurate and robust crRNA design tool (Tables 3, 4). This option enables us to avoid designing crRNA in regions of high structure. Inasmuch as adjacent nucleotides are more likely to interact with each other, target site nucleotides and surrounding 2,000 bases (1,000 upstream and 1,000 downstream) were considered to analyze the secondary structure. The flanking sequences were considered based on the fact that this is unlikely base-pairing interaction among the nucleotides of secondary structure be separated by more than 1,000 nucleotides (Lu and Mathews, 2007). To calculate the probability that each target site is accessible (is not in secondary structure), secondary structures of the target sequences were determined using RNAfold from the ViennaRNA package (Gruber et al., 2008; Lorenz et al., 2011), and a protospacer accessibility value for each target site was computed by dividing the number of target site unpaired bases to the length of target site (0 indicates target site is inaccessible and 1 indicates target site is accessible). Accordingly, frequency of all unpaired bases in 100 nt windows upstream and downstream of spacer site was calculated as local accessibility score. Higher scores indicate that the target sites are more accessible to crRNAs and increase the probability of target site detection.

### 2.8 Self-complementarity

Length and secondary structure (stem-loop structure) of DR sequence in crRNA are highly conserved in different CRISPR-Cas systems, which help Cas protein to recognize the structural features of its cognate crRNA. In this regard, the secondary structure of DR facilitates the interaction of crRNA with Cas protein and is critical for complex formation and its activity (Abudayyeh et al., 2016; Zhang et al., 2018; Wessels et al., 2020). From the experimental data, it is clear that stable base-pairing between DR and spacer can prevent the formation of effector complex, which reduces the efficiency of knockdown functionality of Cas protein (Thyme et al., 2016). Ideally, crRNA sequences to be used in their applications should be the ones that exhibit only natural DR structure. Therefore, in addition to attention to crRNA-target complementarity, potential disruption of DR-spacer interactions should be taken into account. Here, RNA secondary structure along with minimum free energy (MFE) of each potential DR-spacer interactions is predicted using the RNAfold tool to investigate such detrimental interactions. CaSilico reports problematic crRNAs containing DR-spacer interaction as unnatural DR structure, and the predicted structure is displayed in the final output. Users can filter all crRNAs labeled as unnatural DR due to the fact that they may have inhibitory effects on Cas activity. It is essential to note that it is better to behave carefully about unnatural structure of DR, as the interaction of a few bases at the end of the DR with spacer does not destroy the stem secondary structure of DR, which plays a crucial role in target cleavage (Thyme et al., 2016).

### 2.9 Off-target analysis

Specificity is a main factor in using CRISPR-Cas systems in various applications. A perfect crRNA must recognize a specific target site, and no cross-reactivity has to be observed. Since some Cas proteins can tolerate mismatches in spacer-target sequence pairing, the detection of off-target events is undeniable. CaSilico employs the BLASTn tool (with word size = 7 and an E-value = 10 cutoff) to search potential off-target sites for each crRNA candidate within the reference sequence. If the organism of interest is specified by the user, CaSilico considers the genome or transcriptome reference file (based on the type of the Cas protein) of the corresponding organism from reference RNA sequences (refseq_rna) or nucleotide collection (nr/nt) database for off-target analysis. Moreover, users can import a genome or transcriptome reference FASTA file to be considered for off-target prediction analysis. PFS or PAM requirement and the number and position of the mismatches tolerated by the CRISPR-Cas system of interest are taken into account based on the summarized features presented in Tables 3 and 4. Hence, for all Cas systems, only those off-targets with less than or equal to two mismatches are reported as potential off-targets, except type V-A/B in which off-targets are allowed to have ≤4 mismatches at any position. Restriction about the position of mismatch is according to the selected path for identifying conserved windows that will describe the detection of conserved region section. Finally, the potential off-targets are listed in the output file for each crRNA candidate with the following information: chromosome position and number of mismatches. It is obvious that users have to penalize crRNAs that can target the sequence of interest in more than a single site.

### 2.10 Detection of conserved regions

When more than one sequence is given as input, the conserved regions among the sequences are automatically detected by CaSilico and subject to crRNA designing. To this end, first, the sequences are subjected to multiple sequence alignment (MSA) using MAFFT software based on the progressive method (FFT-NS-2) to identify local multiple alignment blocks. Following the MSA, a residue conservation method based on a sliding window size of w (a variable parameter defined by the type of CRISPR-Cas system) is employed across the blocks (stride of 1 nt) to detect the conserved fragments. Our method scans the blocks column-wise and reveals the conservation degree of each position. A conservation score, between 0 and 1, for each position is calculated, as 0 indicates no conservation and 1 indicates full conservation. The MSA and the final consensus sequence files are reported in the result directory. Users are allowed to determine the desired conservation threshold to identify conserved regions (using ConservationThreshold argument, e.g.,, ConservationThreshold = 0.98). There are two different approaches to identify conserved regions: 1) most or all positions in the fragments (with a length of w) have to be conserved and ≤2 positions can be polymorphic (based on the defined conservation score), as these positions can be occurred in the specific locations of the window according to Table 3 and 4 rules and 2) most or all positions in the fragments (with a length of w) have to be conserved and ≤2 positions can be polymorphic (based on the defined conservation score) throughout the fragment (with a length of w) (Abudayyeh et al., 2016, 2017; Cox et al., 2017; Harrington et al., 2018; Tambe et al., 2018; Teng et al., 2018; Zhang et al., 2018; Freije et al., 2019; Wessels et al., 2020). The first method is more conservative and may result in fewer crRNAs than the second method. Finally, the detected conserved fragments are subjected to crRNA designing. In addition to the usual results, for each candidate crRNA that is located in a conserved fragment, position and number of the mismatches, conservation efficiency, and type of detected mismatches are also reported in the table results. Here, mismatches indicate the bases that have a conservation value less than the conservation threshold parameter. Also, conservation efficiency shows the average of conservation values across the detected conserved fragment. Considering the crRNAs in the conserved region with no mismatches and higher conservation efficiency helps to increase the efficiency of the experiment.

### 2.11 Case study and software evaluation

The prevalence of viral diseases in recent years, including SARS-CoV-2, has promoted the World Health Organization (WHO) to declare a global health emergency. To assess the potential of CaSilico to design crRNAs, one case study was performed to design specific crRNAs (for types VI-A/B/D and V-A/B) to target SARS-CoV-2 virus strains. Complete genomes of this virus were retrieved from the GISAID database (Shu and McCauley, 2017). Since huge amounts of SARS-CoV-2 sequences were submitted every day and most of them are similar, especially those that are submitted in close-time proximity, one sequence of each month from each country was considered for analysis. Eventually, 1,412 complete viral genomes without ambiguous nucleotides and indel were obtained. Also, the sequences generated through nonoriginal passages were excluded, as mutations may have occurred during cell culture (Supplementary Material).

Here, the genes that have been used in several SARS-CoV-2 diagnosis protocols including the US Centers for Disease Control and Prevention (CDC), some World Health Organization (WHO) COVID-19 reference laboratories, and SHERLOCK-based and DETECTR-based protocols were considered for crRNA designing (Supplementary Table S2). Also, 3CLPro (3-chymotrypsin-like protease) and nsp8 (nonstructural protein 8) genes that are essential for RNA replication and have not been considered yet for SARS-CoV-2 targeting were investigated (44 and 45) (Supplementary Table S3). To increase the specificity of the designed crRNAs for SARS-CoV-2 detection, off-target analysis for the candidate crRNAs was performed against SARS-CoV and other respiratory viruses, as these viruses are phylogenetically related to SARS-CoV-2 and may affect COVID-19 detection (Supplementary Figure S1; Supplementary Table S4). Moreover, viruses and bacteria with similar clinical presentation (listed in Supplementary Table S5) were considered for off-target analysis. Furthermore, the evaluation of the suggested candidate crRNAs by CaSilico was performed based on gold standard crRNAs, which were previously experimentally tested. Hence, the suggested candidate crRNAs for ten different genes (nsp3, 3CLpro, nsp8, nsp9, nsp10, RdRp, nsp14, S, E, and N) in SARS-CoV-2 genome were compared with those previous studies that experimentally tested crRNAs for these genes (Abbott et al., 2020; Arizti-Sanz et al., 2020; Joung et al., 2020b; Broughton et al., 2020; Zhang et al., 2020).

## 3 Results

**FIGURE 3:**
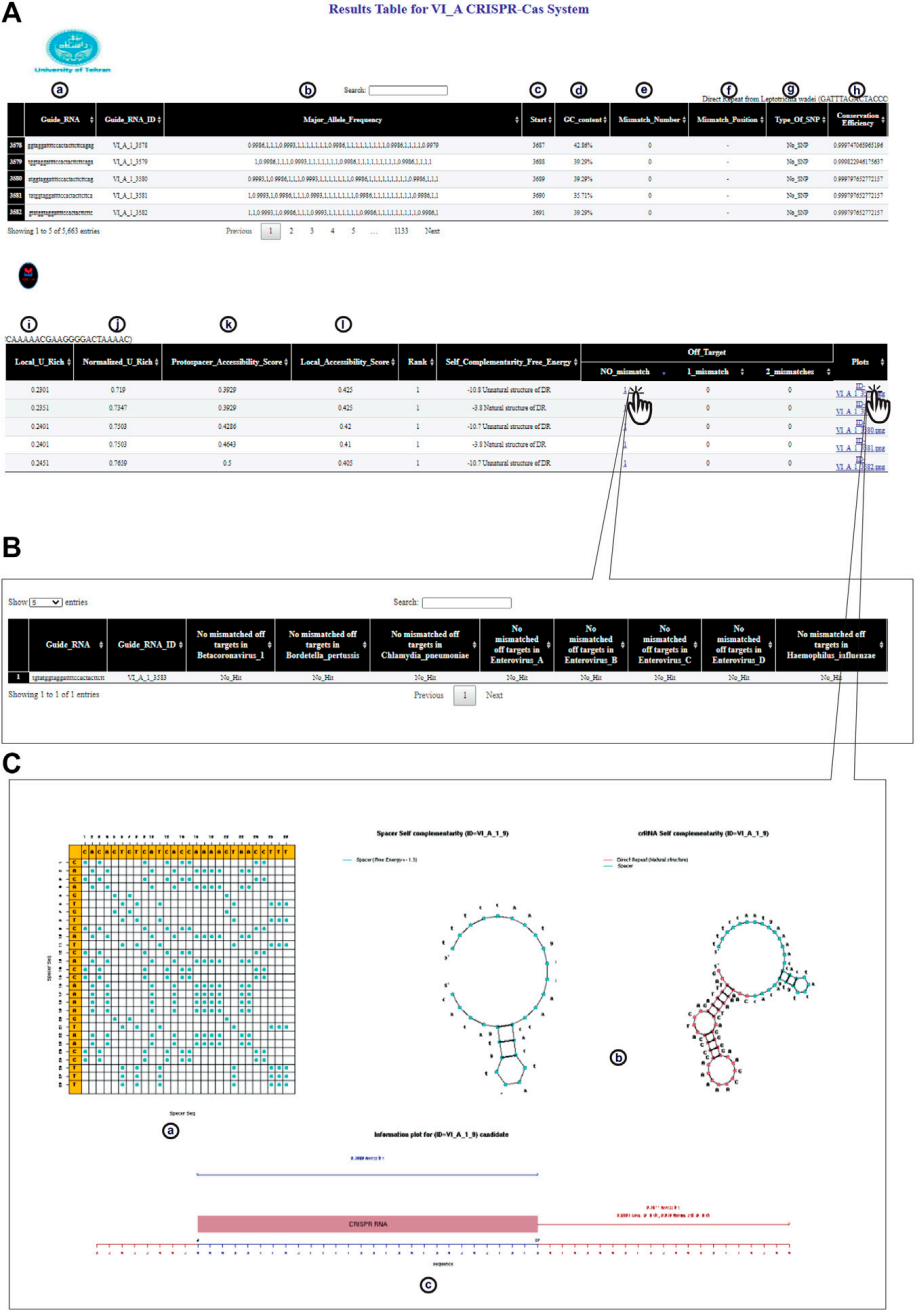
CaSilico represents the results in an interactive table interface. **(A)** Result table shows (a) candidate crRNAs from 5′ to 3', (b) allele frequency for each consensus base all over the window, (c) location of target site on the (consensus) sequence, (d) GC content of the spacer, (e) the number of bases that have an allele frequency less than the conservation threshold, (f) position of each mismatch on the spacer sequence, (g) other bases of a polymorphic position, (h) the average of conservation score of all positions, (i, j) the proportion of Us or As in 100 nt windows upstream and downstream of the spacer target site that is normalized by the proportion of Us or As throughout the gene, (k) the number of target site unpaired bases that are divided by the length of target site, and (l) frequency of all unpaired bases in 100 nt windows upstream and downstream of the target site. **(B)** CaSilico reports all candidate off-targets for inputted organisms by considering some mismatches in another interactive table that is available through clicking of a particular link. Another feature of this table is reporting the gene in which off-target effect is located. **(C)** For each candidate crRNA, CaSilico provides a link in the plots column of the table results and is linked to a graphical view of (a) spacer dot-plot, (b) the predicted self-complementarity of spacer and crRNA, and (c) crRNA information plot.

To facilitate the application of CRISPR-Cas technology and to overcome the limitations of currently available crRNA design tools, CaSilico, a user-friendly R package, was developed to design effective crRNA for user-specified sequence. To make CaSilico more user-friendly, all analysis steps including crRNA designing and off-target searching were integrated into one function “CaSilico.” Moreover, it supports multi-threading to exploit the benefits of multicore architecture. Users only need to provide the information about the target sequence (or set of target sequences) and set the important parameters that help them in selecting the best-performing crRNAs. CaSilico currently supports all the genome/gene/transcript sequences available in the databases, which cover species from yeast to human. However, users are allowed to submit custom sequences to be used for off-target analysis. Finally, all the identified crRNAs in a given input sequence along with their features are displayed in an interactive table as shown in Figure 3. The reported features depend on the type of Cas protein that was selected by the user to design crRNA.

CaSilico currently supports all the genome/gene/transcript sequences available in RefSeq or Ensembl databases, which cover species from yeast to human. However, users are allowed to submit custom genome/gene/transcript sequences to be used for off-target analysis. Finally, all the identified crRNAs in a given input sequence along with their features are displayed in an interactive table as shown in Figure 3. The reported features depend on the type of Cas protein that was selected by the user to design a crRNA. CaSilico benefits the BLAST algorithm to scan a reference sequence (genome or transcriptome) for pattern hits for each crRNA candidate followed by filtering the PAM/PFS-restricted sites and considering the position and number of mismatches. A key point for evaluating the off-target activity of a crRNA is the number of mismatches (Chen et al., 2021). In order to list all the potential off-targets for one crRNA, CaSilico reports all candidate off-targets by considering some mismatches, which is provided in another interactive table and is available through clicking of particular link (Figure 3B). Moreover, for each candidate crRNA, CaSilico provides a link in the plots column of the table results and is linked to a PDF file allowing users to have a graphical view of the predicted secondary structure and other details of that crRNA (Figure 3C). The result tables can be searched, filtered, and re-ordered in terms of various features of crRNAs including GC content, mismatch number, conservation efficiency, protospacer accessibility, and self-complementarity.

To the best of our knowledge, a unique feature of CaSilico is finding the conserved regions among multiple query sequences to be applied for crRNA designing. To showcase this feature, a case study was performed simultaneously on ten genes of SARS-CoV-2 strains to design effective crRNAs that can target these strains (Supplementary Material). A summary of the result of this analysis is shown in Table 5. Evaluation of a crRNA design tool by conducting real experiments is critical in benchmarking the tool to better show the efficiency and usefulness of that tool. Here, to evaluate the performance of CaSilico and test if the designed crRNAs are effective, a benchmark study was performed. Based on the previous studies, the crRNAs designed by CaSilico for the genes in SARS-CoV-2 genome were compared with the tested crRNAs in wet lab experiments. This analysis was performed to provide a demonstration of the functionalities of our design algorithm in CaSilico. Out of the ten genes, for five genes (including nsp3, RdRp, S, E, and N genes), designed crRNAs were identified in the previous studies that belong to VI-A/D and V-A/B subtypes (Abbott et al., 2020; Arizti-Sanz et al., 2020; Broughton et al., 2020; Joung et al., 2020a, 2020b; Zhang et al., 2020). Interestingly, almost all of the experimentally validated crRNAs in the previous studies (41 crRNAs) for the five genes and in different subtypes were predicted by CaSilico. More information about these experimentally validated crRNAs is provided in Supplementary Table S6. Also, out of 5,663; 2,697; 3,539; 7; and 1,409 designed crRNAs, 5,635; 1,888; 3,461; 1; and 856 crRNAs were found to be off-target free for different subtypes of nsp3, RdRp, S, E, and N genes, respectively (Table 5).

**TABLE 5:** Result of crRNA designing for ten genes of SARS-CoV-2 by CaSilico.

Gene subtype			Nsp3	3CLpro	Nsp8	Nsp9	Nsp10	RdRp	Nsp14	S	E	N
VI-A	Candidate		5,663	867	567	312	390	2,636	1,542	3,539	201	1,129
	GC content between 40 and 70		1,293	298	206	176	220	718	550	1,104	67	908
	Local U-rich ≥ 0.25		5,518	867	525	312	390	2,636	1,532	3,526	201	3
	Protospacer accessibility ≥ 0.5		693	114	75	64	69	275	278	692	66	344
	Off-target	No mismatch	9	0	0	16	2	152	3	20	71	149
		One mismatch	12	19	23	13	32	240	27	40	26	69
		Two -mismatches	7	8	8	8	5	53	7	18	2	31
VI-B	Candidate		3,865	580	377	204	252	1745	1,027	2,303	124	622
	GC content between 40 and 70		1,422	312	190	146	174	725	546	1,003	51	544
	Local A-rich ≥ 0.25		3,665	540	376	204	249	1736	978	2059	0	622
	Protospacer accessibility ≥ 0.5		106	68	40	31	44	93	166	253	33	191
	Off-target	No mismatch	2	0	0	6	0	95	0	9	37	64
		One mismatch	5	9	6	4	13	121	13	17	19	37
		Two mismatches	4	8	12	7	4	45	5	19	0	32
VI-D	Candidate		5,730	883	573	318	396	2,697	1,553	3,643	207	1,159
	GC content between 40 and 70		2,103	438	288	217	248	1,131	757	1,591	90	965
	Local U-rich ≥ 0.25		5,591	883	538	316	396	2,697	1,549	3,636	207	9
	Protospacer accessibility ≥ 0.5		828	159	106	78	90	344	335	837	52	369
	Off-target	No mismatch	27	12	8	33	16	325	22	50	87	214
		One mismatch	81	55	70	16	62	420	86	100	33	98
		Two mismatches	16	12	12	6	9	89	18	27	6	28
V-A	Candidate		274	29	21	14	13	118	55	180	7	39
	GC content between 40 and 70		117	16	16	7	9	50	35	93	3	32
	Off-target	No mismatch	0	0	0	0	0	13	2	3	1	10
		One mismatch	3	4	6	0	2	15	3	3	2	3
		Two mismatches	9	1	2	2	2	25	10	13	2	4
		Three mismatches	30	9	3	3	3	27	13	16	1	3
V-B	Candidate		1,268	175	112	52	64	535	267	810	45	211
	GC content between 40 and 70		544	103	65	31	45	242	151	394	24	179
	Off-target	No mismatch	3	2	2	3	0	58	5	17	14	46
		One mismatch	26	18	17	2	16	111	23	27	9	17
		Two mismatches	85	23	22	9	13	140	51	63	10	44
		Three mismatches	274	49	27	17	20	207	77	191	4	36
V-F1	Candidate		5,720	883	575	320	398	2,688	1,554	3,636	209	1,171
	GC content between 40 and 70		2,438	483	312	226	52	1,259	838	1809	105	991
	Protospacer accessibility ≥ 0.5		941	180	114	74	39	411	348	863	52	378
	Off-target	No mismatch	43	20	16	39	0	381	36	74	95	242
		One mismatch	124	54	63	23	6	394	107	122	28	106
		Two mismatches	371	78	50	37	12	385	141	219	10	117

## 4 Discussion

Since its establishment, the CRISPR system has seen a rapid development that reinforces the parallel development of computational algorithms and software to cover the new findings and technologies. In this study, CaSilico was developed to respond to the need for an effective crRNA design tool supporting a wide range of subtypes in class II CRISPR-Cas systems. One of the important advantages of CaSilico is that it is developed as a single modular tool that performs all processes automatically from crRNA identification to off-target analysis. The current version of CaSilico supports either CRISPR/Cas12 (V-A/B/F1 subtypes) or CRISPR/Cas13 (VI-A/B/D subtypes) to design crRNA. It is flexible and can be run with default or user-provided parameters in a range of advanced options governing the design of crRNA. CaSilico supports off-target search in multiple genomes or transcriptome datasets simultaneously. Moreover, our tool can design crRNAs that simultaneously target multiple sequences through conserved region detection among a set of sequences, which is a unique feature in comparison to other tools in this area. The final results are displayed in an interactive table along with a graphical view of the predicted secondary structure and other useful information for each crRNA (Figure 3).

Uniqueness of some features of CaSilico can be better understood if we compare it with the other tools. CHOPCHOP has been developed to design crRNA for Cas9, Cpf1, and Cas13. The user is restricted to choose species proposed by the tool, and only two organisms (*Homo sapiens* and *Mus musculus*) are available for Cas13. CRISPOR permits users to design crRNA for Cas9 and Cpf1 based on the organisms provided by the tool and cannot be used for the organisms out of the list. In this regard, CRISPick has been developed to design crRNA (Cas9 and Cas12a) for human, mouse, and rat. Cas13d tool is dedicated to design crRNA for Cas13; however, it does not consider important features of crRNA designing for Cas13 such as frequency of cleaving base around the target site, mismatch tolerance rules, self-complementarity, target accessibility, and off-target hits, as all of these features are covered by CaSilico for Cas13a/b/d. Moreover, all of the mentioned tools cannot detect conserved regions to design crRNA to target several sequences simultaneously.

CaSilico reports all the potential crRNAs for each user-provided sequence. The key point to be noted is that a crRNA can be very specific, but it is not very efficient or vice versa. Efficiency means the likelihood that the crRNA facilitates precise cutting, and specificity is defined as the likelihood that crRNA binds to the target site of interest. Therefore, a balance between specificity and efficiency has to be taken into account based on the project, as some projects require specificity first and others need more efficiency (Naeem et al., 2020). Hence, CaSilico enables users to have the best choice according to their research purpose, and the provided information can be used to prioritize the validation of the candidate crRNAs. Integration all of the reported features can be applied to help accelerate the process of finding effective crRNAs. Given that some CRISPR-Cas systems such as Cas12 and Cas13 types have recently been developed, the rules governing crRNA design are not completely understood. However, based on the relevant previous studies, users are most advised to consider the conservation and other features such as high specific base content neighboring the crRNA target site and accessibility, which can help them to select a suitable crRNA (Freije et al., 2019; Bandaru et al., 2020). It is worth to note that potential SNPs and RNA editing in the candidate crRNAs should be considered during crRNA designing to promote precise and high-efficient gene editing. As a limitation, CaSilico does not consider these potential variations. However, this issue will be addressed in the next version of the tool.

The viruses usually include multiple strains that are genetically distinct from each other in some species. Also, many genes express different transcript isoforms. Therefore, it is highly desirable to design effective crRNAs for the conserved regions among these sequences to be ensured that all these sequences are covered. But, targeting multi-sequences for simultaneous editing is more difficult than a single sequence. One of the main functionalities that set CaSilico apart from other crRNA design tools is its capability to design crRNAs to target multi-sequences simultaneously. This functionality can be useful to simplify the targeting for viral genome degradation, such as SARS-CoV-2, since there are many thousands of variants of SARS-CoV-2 that differ from each other by at least one mutation. To prove this functionality, CaSilico was applied to design crRNAs targeting different genes in SARS-CoV-2 genome by considering various strains. Results of this analysis highlighted the efficiency of CaSilico to design crRNAs for simultaneous targeting of multi-sequences.

This analysis showed that most of the designed candidates belonged to V-F1 and VI-D subtypes. This finding can be contributed to the point that V-F1 and VI-D subtypes recognize the target sequence without any PAM (PFS) restrictions. Also, they require a short spacer (20 and 22 nt in V-F1 and VI-D, respectively), which is important to increase the number of identified crRNAs (Abudayyeh et al., 2017; Harrington et al., 2018). According to the PAM requirements, V-A subtype with 5′-TTTV PAM showed the lowest number of crRNA candidates. Other subtypes like VI-B and V-B are PAM (PFS) dependent, but a shorter sequence of PAM in these subtypes relative to V-A PAM (5′-DD in VI-B and 5′-TTV in V-B) can lead to find more candidates (Smargon et al., 2017; Teng et al., 2018; Broughton et al., 2020). A determinant factor in the number of designed crRNA candidates is the length of the target sequence. Hence, the highest and lowest numbers of crRNA candidates were found in longest (nsp3) and shortest (E) genes, respectively. Furthermore, the designed crRNAs for SARS-CoV-2 genes were compared with tested crRNAs that were reported in the literature (Supplementary Table S6). Our results showed that CaSilico successfully recommended functional crRNAs for five genes (nsp3, RdRp, S, E, and N) in SARS-CoV-2 genome with acceptable features. For the five genes, apart from the tested crRNAs by the previous studies, additional crRNAs were designed by CaSilico. These candidates along with the designed crRNA candidates for the other five genes (Nsp14, Nsp10, Nsp9, Nsp8, and 3CLpro) showed features comparable to the experimentally validated crRNAs. Hence, these specific crRNAs can be filtered based on different features to choose the best ones for targeting SARS-CoV-2 genes and be considered important candidates for further analysis.

## 5 Conclusion

It is well demonstrated that the efficacy and specificity of a CRISPR-Cas system largely depends on the quality of crRNAs. Here, a Bioconductor package, CaSilico, was developed to facilitate the design of crRNAs for CRISPR-Cas enzymes (Cas12, Cas13, and Cas14) along with off-target analysis in a wide variety of species. Of note, CaSilico supports any reference genome/gene/transcriptome for which a FASTA file is available. Users have the flexibility to modify the processing of crRNA designing through a suitable parameter set such as conservation threshold and the method of identifying conserved windows, allowing users to design an optimal crRNA. CaSilico presents the results in tables and graphs that expedite the quick finding of the most effective candidate crRNAs for the sequence of interest. Depending on the purpose of the study, the best candidate crRNA to target a specific site might not always be the same. Therefore, this tool assists the researchers to find a trade-off between specificity and efficiency of the candidate crRNAs by providing all the important features necessary to identify the best candidate crRNA. In this regard, the users are encouraged to explore the full list of the results. In addition, a functionality was provided to detect conserved regions across the sequences (when more than one sequence needs to be targeted) and target these regions for crRNA designing. This functionality can be useful to simplify the targeting for viral genome degradation, such as SARS-CoV-2, since there are many thousands of variants of SARS-CoV-2 that differ from each other by at least one mutation. In this regard, a bunch of candidate crRNAs targeting important genes in SARS-CoV-2 genome were designed and suggested for future experiments. Our findings validate the prediction accuracy of CaSilico and demonstrate its efficacy for CRISPR-Cas experiments. In the future, we hope to extend the functionality of CaSilico by considering SNPs during crRNA designing and off-target analysis since this strategy can help to have more effective crRNAs.

## Data Availability

The original contributions presented in the study are included in the article/Supplementary Material; further inquiries can be directed to the corresponding author.
