# Supplementary material for: CaSilico: A versatile CRISPR package for in silico CRISPR RNA designing for Cas12, Cas13, and Cas14
Source: Front Bioeng Biotechnol. 2022 Aug 9;10:957131. doi: 10.3389/fbioe.2022.957131 (PMC9395711; doi:10.3389/fbioe.2022.957131)
Supplement: Supplementary file 1 [file DataSheet2.docx]

Supplementary Material

# Supplementary Figures


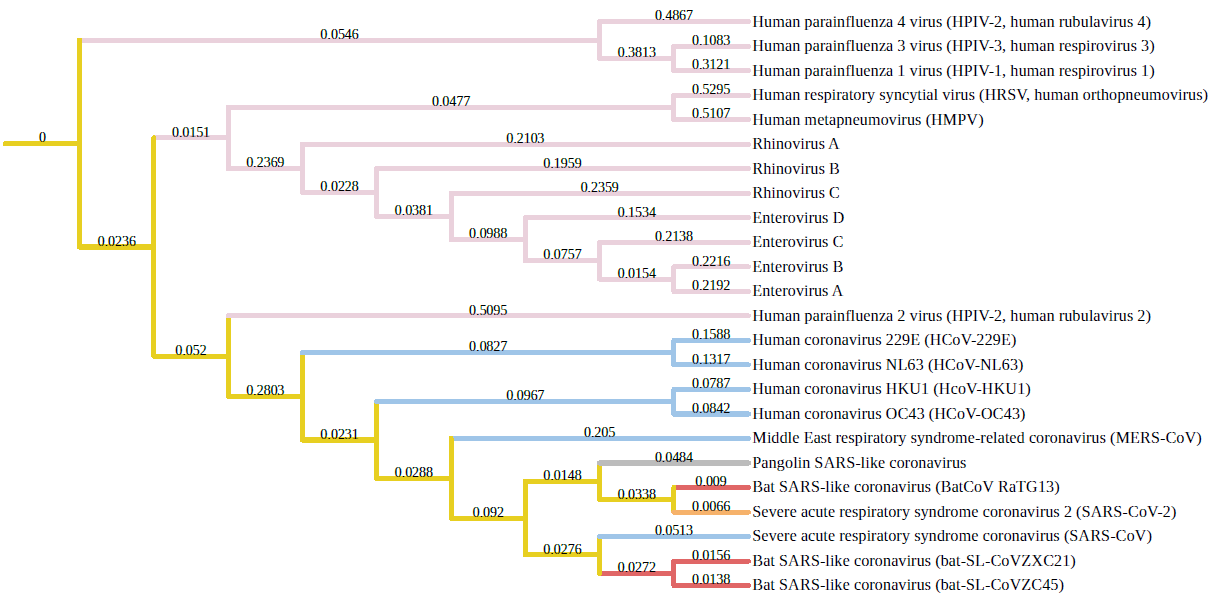


**Supplementary Figure 1.** Genetic relationship among SARS-related coronavirus. This phylogenetic tree shows evolutionary relationships between SARS-CoV-2 and the other viruses including known respiratory viruses, three bat SARS-like coronaviruses, and one pangolin SARS-like coronavirus. The reference genomes of these viruses were obtained from NCBI database (Table S5) and aligned using MAFFT (v7.475) based on the progressive method (FFT‐NS‐2). A phylogenetic tree based on the MSA result was constructed by the neighbor-joining method and jukes-cantor model. Finally, Interactive Tree Of Life (iTOL, v6) platform was used for further phylogenetic tree visualization and annotation. Labels show the name of each virus.

# Supplementary Tables

**Supplementary Table 1.** The list of 18 crRNA design tools (Chuai et al., 2017; Hwang et al., 2021).

| **Tool** | **Cas enzymes** | **Species support** | **Supported PAM** | **off-target search** | **URL** |
| --- | --- | --- | --- | --- | --- |
| **Cas-Designer** | Cas9 and Cas12 variants | Many | a variety of PAM sequences | Yes | http://www.rgenome.net/cas-designer/ |
| **CRISPR-P 2.0** | Cas9 and Cas12 variants | Many | a variety of PAM sequences | Yes | http://crispr.hzau.edu.cn/CRISPR2/ |
| **CRISPRdirect** | Cas9 | Many | a variety of PAM sequences | Yes | https://crispr.dbcls.jp/ |
| **CCTop** | Cas9 and Cas12 variants | Many | a variety of PAM sequences | Yes | http://crispr.cos.uni-heidelberg.de/ |
| **DeepCpf1** | AsCpf1 | a variety of cell lines | TTTV | No | http://deepcrispr.info/ |
| **DeepSpCas9** | SpCas9 | Many | NGG | No | http://deepcrispr.info/DeepSpCas9 |
| **DeepHF** | SpCas9  variants | Many | NGG | No | http://www.DeepHF.com/ |
| **CINDEL** | AsCpf1 | Many | TTTV | No | http://big.hanyang.ac.kr/cindel |
| **SSC** | SpCas9 | Many | NGG | No | http://cistrome.org/SSC/ |
| **CRISPRscan** | Cas9 and Cas12 variants | Many | a variety of PAM sequences | Yes | https://www.crisprscan.org |
| **sgRNAcas9** | Cas9 | Many | NGG | Yes | http://www.biootools.com/ |
| **CasFinder** | Cas9 variants | Human, mouse | a variety of PAM sequences | Yes | http://arep.med.harvard.edu/CasFinder/ |
| **CRISPR-ERA** | Cas9 | Many | NGG | Yes | http://crispr-era.stanford.edu/ |
| **SgRNA Scorer** | Cas9 and Cas12 variants | Many | a variety of PAM sequences | Yes | http://crispr.med.harvard.edu/sgRNAScorer |
| **CRISPR**  **multitargeter** | Cas9 variants | Many | a variety of PAM sequences | Yes | http://www.multicrispr.net/ |
| **WU-CRISPR** | Cas9 | Many | NGG | Yes | http://crispr.wustl.edu/ |
| **CRISPR Library**  **Designer (CLD)** | Cas9 variants | Many | a variety of PAM sequences | Yes | https://github.com/boutroslab/cld |
| **CRISPR-RT** | Cas13a | Many | a variety of PFS sequences | Yes | http://bioinfolab.miamioh.edu/CRISPR-RT/index.php |

**Supplementary Table 2.** Validated real-time RT-PCR amplicons or CRISPR-Cas system spacers of SARS-CoV-2 diagnostic protocols. These protocols have been recommended by various publications.

| Publication | Explanation |
| --- | --- |
| China CDC Primers and probes for detection 2019-nCoV^1^ | Amplicon ORF1ab of the SARS-CoV-2 real-time RT-PCR diagnostic panel |
|  | 5′- CCCTGTGGGTTTTACACTTAAAAACACAGTCTGTACCGTCTGCGGTATGTGGAAAGGTTA  TGGCTGTAGTTGTGATCAACTCCGCGAACCCATGCTTCAGTCAGCTGATGCACAATCGT -3′ |
|  | Amplicon N of the SARS-CoV-2 real-time RT-PCR diagnostic panel |
|  | 5′- GGGGAACTTCTCCTGCTAGAATGGCTGGCAATGGCGGTGATGCTGCTCTTGCTTTGCTGC  TGCTTGACAGATTGAACCAGCTTGAGAGCAAAATGTCTG -3′ |
| Detection of 2019 novel coronavirus (2019-nCoV) in suspected human cases by RT-PCR (HKU)^2^ | Amplicon ORF1b-nsp14 of the SARS-CoV-2 real-time RT-PCR diagnostic panel |
|  | 5′- TGGGGTTTTACAGGTAACCTACAAAGCAACCATGATCTGTATTGTCAAGTCCATGGTAATGCACA  TGTAGCTAGTTGTGATGCAATCATGACTAGGTGTCTAGCTGTCCACGAGTGCTTTGTTAAGCGTGTT -3′ |
|  | Amplicon N of the SARS-CoV-2 real-time RT-PCR diagnostic panel |
|  | 5′- TAATCAGACAAGGAACTGATTACAAACATTGGCCGCAAATTGCACAATTTGCCCCCAGCG  CTTCAGCGTTCTTCGGAATGTCGCGCATTGGCATGGAAGTCACACCTTCG -3′ |
| Diagnostic detection of Wuhan coronavirus 2019 by real-time RT-PCR – Charité, Berlin Germany (Corman et al., 2020)^3^ | Amplicon RdRp of the SARS-CoV-2 real-time RT-PCR diagnostic panel |
|  | 5′- GTGAAATGGTCATGTGTGGCGGTTCACTATATGTTAAACCAGGTGGAACCTCATCAGGA  GATGCCACAACTGCTTATGCTAATAGTGTTTTTAACATTTG -3′ |
|  | Amplicon E of the SARS-CoV-2 real-time RT-PCR diagnostic panel |
|  | 5′- ACAGGTACGTTAATAGTTAATAGCGTACTTCTTTTTCTTGCTTTCGTGGTATTCTTGCTAG  TTACACTAGCCATCCTTACTGCGCTTCGATTGTGTGCGTACTGCTGCAATAT -3′ |
| US CDC panel primers and probes – U.S. CDC, USA^4^ | Amplicon N1 of the SARS-CoV-2 real-time RT-PCR diagnostic panel |
|  | 5′- GACCCCAAAATCAGCGAAATGCACCCCGCATTACGTTTGGTGGACCCTCAGATTCAACTG  GCAGTAACCAGA -3′ |
|  | Amplicon N2 of the SARS-CoV-2 real-time RT-PCR diagnostic panel |
|  | 5′- TTACAAACATTGGCCGCAAATTGCACAATTTGCCCCCAGCGCTTCAGCGTTCTTCGGAATG  TCGCGC -3′ |
| PCR and sequencing protocol for 2019-nCoV - Ministry of Public Health, Thailand^5^ | Amplicon N of the SARS-CoV-2 real-time RT-PCR diagnostic panel |
|  | 5′- CGTTTGGTGGACCCTCAGATTCAACTGGCAGTAACCAGAATGGAGAACGCAGTGGGG -3′ |
| Real-time RT-PCR assays for the detection of SARS-CoV 2 Institut Pasteur, Paris^6^ | Amplicon ORF1ab-nsp9 of the SARS-CoV-2 real-time RT-PCR diagnostic panel |
|  | 5′- ATGAGCTTAGTCCTGTTGCACTACGACAGATGTCTTGTGCTGCCGGTACTACACAAACTG  CTTGCACTGATGACAATGCGTTAGCTTACTACAACACAACAAAGGGAG -3′ |
|  | Amplicon RdRp of the SARS-CoV-2 real-time RT-PCR diagnostic panel |
|  | 5′- GGTAACTGGTATGATTTCGGTGATTTCATACAAACCACGCCAGGTAGTGGAGTTCCTGTT  GTAGATTCTTATTATTCATTGTTAATGCCTATATTAACCTTGACCAG -3′ |
|  | Amplicon E of the SARS-CoV-2 real-time RT-PCR diagnostic panel |
|  | 5′- ACAGGTACGTTAATAGTTAATAGCGTACTTCTTTTTCTTGCTTTCGTGGTATTCTTGCTAG  TTACACTAGCCATCCTTACTGCGCTTCGATTGTGTGCGTACTGCTGCAATAT -3′ |
| A protocol for detection of COVID-19 using CRISPR diagnostics^7^ | LwaCas13a spacer for detecting S gene by SHERLOCK technique |
|  | 5′- GCAGCACCAGCTGTCCAACCTGAAGAAG -3′ |
|  | Target genomic region |
|  | 5′- CTTCTTCAGGTTGGACAGCTGGTGCTGC -3′ |
|  | LwaCas13a spacer for detecting Orf1ab gene by SHERLOCK technique |
|  | 5′- CCAACCTCTTCTGTAATTTTTAAACTAT -3′ |
|  | Target genomic region |
|  | 5′- ATAGTTTAAAAATTACAGAAGAGGTTGG -3′ |
| CRISPR-based surveillance for COVID-19 using genomically-comprehensive machine learning design^8^ | LwaCas13a spacer for detecting Orf1ab gene by SHERLOCK technique |
|  | 5′- CTCTTCTTCAGGTTGAAGAGCAGCAGAA -3′ |
|  | Target genomic region |
|  | 5′- TTCTGCTGCTCTTCAACCTGAAGAAGAG -3′ |
| point-of-care testing for covid-19 using sherlock diagnostics (Arizti-Sanz et al., 2020)^9^ | AaCas12b spacer for detecting N gene by SHERLOCK technique |
|  | 5′- CGAAGAACGCTGAAGCGCTG -3′ |
|  | Target genomic region |
|  | 5′- CAGCGCTTCAGCGTTCTTCG -3′ |
| A protocol for rapid detection of the 2019 novel coronavirus SARS-CoV-2 using CRISPR diagnostics: SARS-CoV-2 DETECTR (Broughton et al., 2020)^10^ | LbCas12a spacer for detecting N gene by DETECTR technique (specific to SARS-CoV-2, compatible with same N2 amplicon region as in the CDC protocol) |
|  | 5′- CCCCCAGCGCTTCAGCGTTC -3′ |
|  | Target genomic region |
|  | 5′- CCCCCAGCGCTTCAGCGTTC -3′ |
|  | LbCas12a spacer for detecting E gene by DETECTR technique (specific to pan-coronavirus, compatible with same amplicon region as in the WHO protocol) |
|  | 5′- GTGGTATTCTTGCTAGTTAC -3′ |
|  | Target genomic region |
|  | 5′- GTGGTATTCTTGCTAGTTAC -3′ |

1. (http://ivdc.chinacdc.cn/kyjz/202001/t20200121_211337.html)
2. (https://www.who.int/docs/default-source/coronaviruse/peiris-protocol-16-1-20.pdf?sfvrsn=af1aac73_4)
3. (https://www.who.int/docs/default-source/coronaviruse/protocol-v2-1.pdf?sfvrsn=a9ef618c_2)
4. (https://www.who.int/docs/default-source/coronaviruse/uscdcrt-pcr-panel-primer-probes.pdf?sfvrsn=fa29cb4b_2)
5. https://www.who.int/docs/default-source/coronaviruse/conventional-rt-pcr-followed-by-sequencing-for-detection-of-ncov-rirl-nat-inst-health-t.pdf?sfvrsn=42271c6d_4
6. https://www.who.int/docs/default-source/coronaviruse/real-time-rt-pcr-assays-for-the-detection-of-sars-cov-2-institut-pasteur-paris.pdf?sfvrsn=3662fcb6_2
7. https://www.broadinstitute.org/files/publications/special/COVID-19%20detection%20(updated).pdf
8. https://www.biorxiv.org/content/10.1101/2020.02.26.967026v2.full.pdf
9. https://www.medrxiv.org/content/10.1101/2020.05.04.20091231v1.full.pdf
10. https://mammoth.bio/wp-content/uploads/2020/03/Mammoth-Biosciences-A-protocol-for-rapid-detection-of-SARS-CoV-2-using-CRISPR-diagnostics-DETECTR.pdf

**Supplementary Table 3.** List of genes encoded by SARS-CoV-2 genome according to the NCBI reference genome.

| **Gene** | **Protein product** | **Protein type** | **Location** | **Length (bp)** |
| --- | --- | --- | --- | --- |
| **ORF1ab** | ORF1ab polyprotein | NSP^*^ | 266-21555 | 21290 |
| **ORF1ab** | nsp1 (leader protein) | NSP | 266-805 | 540 |
| **ORF1ab** | nsp2 | NSP | 806-2719 | 1914 |
| **ORF1ab** | nsp3 | NSP | 2720-8554 | 5835 |
| **ORF1ab** | nsp4 | NSP | 8555-10054 | 1500 |
| **ORF1ab** | 3C-like protease (3CL^pro^) | NSP | 10055-10972 | 918 |
| **ORF1ab** | nsp6 | NSP | 10973-11842 | 870 |
| **ORF1ab** | nsp7 | NSP | 11843-12091 | 249 |
| **ORF1ab** | nsp8 | NSP | 12092-12685 | 594 |
| **ORF1ab** | nsp9 (ssRNA-binding protein) | NSP | 12686-13024 | 339 |
| **ORF1ab** | nsp10 | NSP | 13025-13441 | 417 |
| **ORF1ab** | nsp11 | NSP | 13442-13480 | 39 |
| **ORF1ab** | nsp12 (RNA-dependent RNA polymerase, RdRp) | NSP | join(13442-13468,13468-16236) | 2795 |
| **ORF1ab** | helicase | NSP | 16237-18039 | 1803 |
| **ORF1ab** | 3' to 5' exonuclease | NSP | 18040-19620 | 1581 |
| **ORF1ab** | endoRNAse | NSP | 19621-20658 | 1038 |
| **ORF1ab** | 2'-O-ribose methyltransferase | NSP | 20659-21552 | 894 |
| **S (spike glycoprotein)** | surface glycoprotein | SP^**^ | 21563-25384 | 3822 |
| **ORF3a** | ORF3a protein | NSP | 25393-26220 | 828 |
| **E** | envelope protein | SP | 26245-26472 | 228 |
| **M** | membrane glycoprotein | SP | 26523-27191 | 669 |
| **ORF6** | ORF6 protein | NSP | 27202-27387 | 186 |
| **ORF7a** | ORF7a protein | NSP | 27394-27759 | 366 |
| **ORF7b** | ORF7b | NSP | 27756-27887 | 132 |
| **ORF8** | ORF8 protein | NSP | 27894-28259 | 366 |
| **N** | nucleocapsid phosphoprotein | SP | 28274-29533 | 1260 |
| **ORF10** | ORF10 protein | NSP | 29558-29674 | 117 |

^*^ Non-Structural Protein, ^**^ Structural Protein

**Supplementary Table 4.** The full genome sequences used for creating the phylogenetic tree of SARS-related coronavirus.

| **Virus Name** | **Accession Number** |
| --- | --- |
| **Severe acute respiratory syndrome coronavirus 2 (SARS-CoV-2)** | NC045512 |
| **Severe acute respiratory syndrome coronavirus (SARS-CoV)** | NC004718 |
| **Middle East respiratory syndrome coronavirus (MERS-CoV)** | NC019843 |
| **Human coronavirus 229E (HCoV-229E)** | NC002645 |
| **Human coronavirus OC43 (HCoV-OC43)** | NC006213 |
| **Human coronavirus NL63 (HCoV-NL63)** | NC005831 |
| **Human coronavirus HKU1 (HCoV-HKU1)** | NC006577 |
| **Bat SARS-like coronavirus (bat-SL-CoVZC45)** | MG772933 |
| **Bat SARS-like coronavirus (bat-SL-CoVZXC21)** | MG772934 |
| **Bat SARS-like coronavirus (BatCoV RaTG13)** | MN996532 |
| **Pangolin SARS-like coronavirus** | MT084071 |
| **Human parainfluenza 1 virus (HPIV-1, human respirovirus 1)** | NC003461 |
| **Human parainfluenza 2 virus (HPIV-2, human rubulavirus 2)** | NC003443 |
| **Human parainfluenza 3 virus (HPIV-3, human respirovirus 3)** | NC001796 |
| **Human parainfluenza 4 virus (HPIV-2, human rubulavirus 4)** | NC021928 |
| **Rhinovirus A** | NC001617 |
| **Rhinovirus B** | NC001490 |
| **Rhinovirus C** | NC009996 |
| **Enterovirus A** | NC001612 |
| **Enterovirus B** | NC001472 |
| **Enterovirus C** | NC002058 |
| **Enterovirus D** | NC001430 |
| **Human respiratory syncytial virus (HRSV, human orthopneumovirus)** | NC001781 |
| **Human metapneumovirus (HMPV)** | NC039199 |

**Supplementary Table 5.** The SARS-CoV-2 spacers were blasted against the phylogenetically-related viruses to SARS-CoV-2, viruses, and bacteria with similar clinical presentation listed below.

| **Virus or bacterium name** | **Taxid** |
| --- | --- |
| **Severe acute respiratory syndrome coronavirus (SARS-CoV)** | 694009 |
| **Middle East respiratory syndrome coronavirus (MERS-CoV)** | 1335626 |
| **Human coronavirus 229E (HCoV-229E)** | 11137 |
| **Betacoronavirus 1** | 694003 |
| **Human coronavirus OC43 (HCoV-OC43)** | 31631 |
| **Human coronavirus NL63 (HCoV-NL63)** | 277944 |
| **Human coronavirus HKU1 (HCoV-HKU1)** | 290028 |
| **Influenza A virus** | 11320 |
| **Influenza B virus** | 11520 |
| **Human parainfluenza 1 virus (HPIV-1, human respirovirus 1)** | 12730 |
| **Human parainfluenza 2 virus (HPIV-2, human rubulavirus 2)** | 2560525 |
| **Human parainfluenza 3 virus (HPIV-3, human respirovirus 3)** | 11216 |
| **Human parainfluenza 4 virus (HPIV-4, human rubulavirus 4)** | 2560526 |
| **Rhinovirus A** | 147711 |
| **Rhinovirus B** | 147712 |
| **Rhinovirus C** | 463676 |
| **Enterovirus A** | 138948 |
| **Enterovirus B** | 138949 |
| **Enterovirus C** | 138950 |
| **Enterovirus D** | 138951 |
| **Human respiratory syncytial virus (HRSV, human orthopneumovirus)** | 11250 |
| **Human metapneumovirus (HMPV)** | 162145 |
| **Human bocavirus** | 329641 |
| **Legionella** | 445 |
| **Mycoplasma** | 2093 |
| **Haemophilus influenzae** | 727 |
| **Klebsiella pneumoniae** | 573 |
| **Streptococcus pneumoniae** | 1313 |
| **Pseudomonas aeruginosa** | 287 |
| **Staphylococcus aureus** | 1280 |
| **Chlamydia pneumoniae** | 83558 |
| **Bordetella pertussis** | 520 |

**Supplementary Table 6.** Prediction of experimentally validated crRNAs in different studies by Casilico along with important features of these crRNAs.

| **Guide-RNA** (5′-3′) | **Target Gene** | **System** | **Mismatch­Number** | **Local-U-Rich** | **Normalized-U-Rich** | **Protospacer­Accessibility­Score** | **Local­-Accessibility-Score** | **Self­­­­-Complementarity** | **Reference** |
| --- | --- | --- | --- | --- | --- | --- | --- | --- | --- |
| GCAGCACCAGCTGTCCAACCTGAAGAAG | S | VI-A | 0 | 0.3601 | 1.0826 | 0.4643 | 0.305 | Unnatural structure of DR | (Zhang et al., 2020) |
| CCAACCTCTTCTGTAATTTTTAAACTAT | Nsp3 | VI-A | 0 | 0.2801 | 0.8753 | 0.8214 | 0.355 | Unnatural structure of DR^**^ |  |
| CTCTTCTTCAGGTTGAAGAGCAGCAGAA | Nsp3 | VI-A | 0 | 0.2402 | 0.7506 | 0.3571 | 0.295 | Natural structure of DR | (Arizti-Sanz et al., 2020) |
| CGAAGAACGCTGAAGCGCTG | N | V-B | 0 | NA^*^ | NA | NA | NA | Natural structure of DR | (Joung et al., 2020) |
| CCCCCAGCGCTTCAGCGTTC | N | V-A | 0 | NA | NA | NA | NA | Natural structure of DR | (Broughton et al., 2020) |
| GTGGTATTCTTGCTAGTTAC | E | V-A | 0 | NA | NA | NA | NA | Natural structure of DR |  |
| TCCTGAGCAAAGAAGAAGTGTT | RdRp | VI-D | 0 | 0.3603 | 1.109 | 0.3636 | 0.35 | Unnatural structure of DR | (Abbott et al., 2020) |
| TGTCTGATATCACACATTGTTG | RdRp | VI-D | 0 | 0.3551 | 1.093 | 0.3636 | 0.345 | Unnatural structure of DR |  |
| TCTGATCCCAATATTTAAAATA | RdRp | VI-D | 0 | 0.3346 | 1.0299 | 0.0909 | 0.37 | Natural structure of DR |  |
| GTCTCTTAACTACAAAGTAAGA | RdRp | VI-D | 0 | 0.3301 | 1.0161 | 0.3182 | 0.34 | Natural structure of DR |  |
| GGCGTACACGTTCACCTAAGTT | RdRp | VI-D | 0 | 0.3201 | 0.9853 | 0.4091 | 0.31 | Unnatural structure of DR |  |
| GATTCATTTGAGTTATAGTAGG | RdRp | VI-D | 0 | 0.3249 | 1.0001 | 0.1818 | 0.31 | Natural structure of DR |  |
| CTTGAGCACACTCATTAGCTAA | RdRp | VI-D | 0 | 0.3201 | 0.9853 | 0.2727 | 0.32 | Natural structure of DR |  |
| TAGCAGGGTCAGCAGCATACAC | RdRp | VI-D | 0 | 0.3352 | 1.0318 | 0.4091 | 0.315 | Unnatural structure of DR |  |
| AAACATTAAAGTTTGCACAATG | RdRp | VI-D | 0 | 0.3396 | 1.0453 | 0.3182 | 0.325 | Unnatural structure of DR |  |
| GACAACAATTAGTTTTTAGGAA | RdRp | VI-D | 0 | 0.2801 | 0.8622 | 0.0455 | 0.365 | Unnatural structure of DR |  |
| ATATATGTGGTACCATGTCACC | RdRp | VI-D | 0 | 0.3101 | 0.9545 | 0.4091 | 0.335 | Natural structure of DR |  |
| ATTACCTTCATCAAAATGCCTT | RdRp | VI-D | 0 | 0.315 | 0.9696 | 0.4091 | 0.315 | Unnatural structure of DR |  |
| CTTGATTATCTAATGTCAGTAC | RdRp | VI-D | 0 | 0.3451 | 1.0622 | 0.3182 | 0.315 | Natural structure of DR |  |
| AAGAATCTACAACAGGAACTCC | RdRp | VI-D | 0 | 0.3251 | 1.0007 | 0.1364 | 0.35 | Natural structure of DR |  |
| AGCAAAATTCATGAGGTCCTTT | RdRp | VI-D | 0 | 0.3201 | 0.9853 | 0.2727 | 0.35 | Natural structure of DR |  |
| AACATTTTGCTTCAGACATAAA | RdRp | VI-D | 0 | 0.3251 | 1.0007 | 0.1818 | 0.37 | Unnatural structure of DR |  |
| CACTATTAGCATAAGCAGTTGT | RdRp | VI-D | 0 | 0.3101 | 0.9545 | 0.3636 | 0.365 | Natural structure of DR |  |
| TTGAATCTGAGGGTCCACCAAA | N | VI-D | 0 | 0.1889 | 0.8968 | 0.1818 | 0.3087 | Unnatural structure of DR |  |
| CCCCACTGCGTTCTCCATTCTG | N | VI-D | 0 | 0.2209 | 1.0487 | 0.0909 | 0.3571 | Natural structure of DR |  |
| TGAACCAAGACGCAGTATTATT | N | VI-D | 0 | 0.1908 | 0.9058 | 0.3636 | 0.39 | Unnatural structure of DR^**^ |  |
| AACGCCTTGTCCTCGAGGGAAT | N | VI-D | 0 | 0.2007 | 0.9528 | 0.4545 | 0.445 | Natural structure of DR |  |
| GGTAGCTCTTCGGTAGTAGCCA | N | VI-D | 0 | 0.2308 | 1.0957 | 0.3636 | 0.41 | Natural structure of DR |  |
| AGGCTCCCTCAGTTGCAACCCA | N | VI-D | 0 | 0.22 | 1.0444 | 0.2273 | 0.5 | Unnatural structure of DR^**^ |  |
| AGGATTGCGGGTGCCAATGTGA | N | VI-D | 0 | 0.1907 | 0.9053 | 0.5455 | 0.445 | Unnatural structure of DR |  |
| TTTTGGCAATGTTGTTCCTTGA | N | VI-D | 0 | 0.2101 | 0.9974 | 0.6818 | 0.425 | Natural structure of DR |  |
| GAAGAGGCTTGACTGCCGCCTC | N | VI-D | 0 | 0.2168 | 1.0292 | 0.3636 | 0.43 | Natural structure of DR |  |
| GCCTCAGCAGCAGATTTCTTAG | N | VI-D | 0 | 0.211 | 1.0017 | 0.2727 | 0.36 | Unnatural structure of DR |  |
| GTGGCAGTACGTTTTTGCCGAG | N | VI-D | 0 | 0.1802 | 0.8555 | 0.2727 | 0.4 | Unnatural structure of DR |  |
| CTTGGGTTTGTTCTGGACCACG | N | VI-D | 0 | 0.215 | 1.0207 | 0.4091 | 0.38 | Natural structure of DR |  |
| AATCAGTTCCTTGTCTGATTAG | N | VI-D | 0 | 0.2001 | 0.9499 | 0.3182 | 0.395 | Natural structure of DR |  |
| AAATTGTGCAATTTGCGGCCAA | N | VI-D | 0 | 0.2 | 0.9495 | 0.3182 | 0.41 | Unnatural structure of DR |  |
| CCACGTTCCCGAAGGTGTGACT | N | VI-D | 0 | 0.245 | 1.1631 | 0.4091 | 0.455 | Unnatural structure of DR |  |
| TTTGGATCTTTGTCATCCAATT | N | VI-D | 0 | 0.2159 | 1.0249 | 0.4545 | 0.48 | Natural structure of DR |  |
| GAATGTTTTGTATGCGTCAATA | N | VI-D | 0 | 0.1902 | 0.9029 | 0.6818 | 0.445 | Unnatural structure of DR |  |
| TCTTCTTTTTGTCCTTTTTAGG | N | VI-D | 0 | 0.2352 | 1.1166 | 0.5 | 0.445 | Natural structure of DR |  |

^*^ Not applicable, ^**^ Stem secondary structure of DR is damaged.

**References**

Abbott, T. R., Dhamdhere, G., Liu, Y., Lin, X., Goudy, L., Zeng, L., et al. (2020). Development of CRISPR as an Antiviral Strategy to Combat SARS-CoV-2 and Influenza. *Cell* 181, 865-876.e12. doi: 10.1016/j.cell.2020.04.020.

Arizti-Sanz, J., Freije, C. A., Stanton, A. C., Petros, B. A., Boehm, C. K., Siddiqui, S., et al. (2020). Streamlined inactivation, amplification, and Cas13-based detection of SARS-CoV-2. *Nat. Commun.* 11, 5921. doi: 10.1038/s41467-020-19097-x.

Broughton, J. P., Deng, X., Yu, G., Fasching, C. L., Servellita, V., Singh, J., et al. (2020). CRISPR–Cas12-based detection of SARS-CoV-2. *Nat. Biotechnol.* 38, 870–874. doi: 10.1038/s41587-020-0513-4.

Chuai, G., Wang, Q.-L., and Liu, Q. (2017). In Silico Meets In Vivo : Towards Computational CRISPR-Based sgRNA Design. *Trends Biotechnol.* 35, 12–21. doi: 10.1016/j.tibtech.2016.06.008.

Corman, V. M., Landt, O., Kaiser, M., Molenkamp, R., Meijer, A., Chu, D. K., et al. (2020). Detection of 2019 novel coronavirus (2019-nCoV) by real-time RT-PCR. *Eurosurveillance* 25. doi: 10.2807/1560-7917.ES.2020.25.3.2000045.

Hwang, G.-H., Song, B., and Bae, S. (2021). Current widely-used web-based tools for CRISPR nucleases, base editors, and prime editors. *Gene Genome Ed.* 1, 100004. doi: 10.1016/j.ggedit.2021.100004.

Joung, J., Ladha, A., Saito, M., Segel, M., Bruneau, R., Huang, M., et al. (2020). Point-of-care testing for COVID-19 using SHERLOCK diagnostics. *medRxiv Prepr. Serv. Heal. Sci.* doi: 10.1101/2020.05.04.20091231.

Zhang, F., Abudayyeh, O. O., Gootenberg, J. S., Sciences, C., and Mathers, L. (2020). A protocol for detection of COVID-19 using CRISPR diagnostics. *Bioarchive*, 1–8.
